# A proteomic analysis of the endometrium in obese and overweight women with recurrent miscarriage: preliminary evidence for an endometrial defect

**DOI:** 10.1186/1477-7827-12-75

**Published:** 2014-08-05

**Authors:** Mostafa Metwally, Rebecca Preece, Jerry Thomas, William Ledger, Tin Chiu Li

**Affiliations:** 1 Department of Obstetrics and Gynaecology, The Jessop Wing and Royal Hallamshire Hospital, University of Sheffield, Sheffield S10 2SF, UK; 2 Department of Biology, Technology Facility, University of York, York, UK; 3 Women’s & Children’s Department, Royal Hospital for Women, University of New South Wales, Kensington, Paddington, NSW, Australia; 4 Department of Obstetrics and Gynaecology, The Jessop Wing and Royal Hallamshire Hospital, Sheffield S10 2SF, UK

**Keywords:** Obesity, Miscarriage, Endometrium, Proteomics

## Abstract

**Background:**

Overweight and obese women have been shown to have an increased risk of recurrent miscarriage as well as other adverse reproductive outcomes, but it is yet unclear whether this is due to an effect on the endometrium, embryo or both. The current study employs proteomic analysis to examine for a potential endometrial defect in obese and overweight women with recurrent miscarriage.

**Methods:**

Proteomic tissue analysis of 21 endometrial samples obtained In the midluteal phase from 16 women with recurrent miscarriage (obese, n = 12 and lean, n = 4) and 5 fertile volunteers (Obese, n = 2 and Lean, n = 3). Proteins were separated using 2-D gel electrophoresis and principle component analysis was used to quantitatively compare protein expression between groups. Protein spots showing significantly altered expression were identified using mass spectrometry.

**Results:**

Obese and overweight recurrent miscarriage patients had a significantly increased endometrial expression of haptoglobin compared to their lean counterparts (p = 0.01). These patients also displayed a significant increase in endometrial expression of transthyretin (p = 0.04) and beta- globulin (p = 0.04). Principle Component Analysis (PCA) of the studied groups also demonstrated that endometrial samples could be grouped based on differences in the BMI, suggesting that obesity is an independent factor influencing endometrial protein expression.

**Conclusions:**

These findings provide preliminary evidence for an alteration in the endometrial protein profile in overweight/obese women with recurrent miscarriage mainly in the form of increased haptoglobin, an inflammatory marker associated with obesity.

## Background

There is growing evidence that an increase in body mass index (BMI) is associated an increased risk of miscarriage [1-3]. It is however unknown if this association is due to an adverse effect on the embryo, endometrium or both.

The possibility of an endometrial defect was first postulated in a study of assisted conception cycles using the oocyte donation model [4]. In an earlier study, we attempted to identify the nature of any possible endometrial defect by examining several markers of endometrial function, including endometrial morphology, leukocyte populations, leukaemia inhibitory factor and steroid receptors using immunocytochemical staining, but were unable to identify any clear effect on any of these factors [5].

However there are a vast number of endometrial factors involved in the implantation process and it is possible that a specific endometrial defect was missed. To prospectively examine all possible endometrial factors using immunocytochemical staining or other molecular techniques could be a time consuming and possibly unrevealing process, so we postulated that by mapping the endometrial protein profile around the time of implantation, potential target proteins could be identified for further investigation.

The aim of this study was to determine firstly if overweight and obesity are associated with a change in the endometrial protein profile and secondly if such a change could reflect an endometrial cause for the increased risk of miscarriage in overweight and obese women.

## Methods

### Study population

Endometrial samples from 16 consecutive women suffering from recurrent miscarriage and consenting to participation were included as the study group. The study was conducted at the recurrent miscarriage clinic of the Jessop Wing, Royal Hallamshire Hospital, Sheffield, UK. Recurrent miscarriage was defined as the occurrence of three or more consecutive miscarriages at less than 20 weeks gestation. For each participant, the BMI was calculated from the formula; weight (kg)/height^2^ (m^2^). A normal BMI (lean group) was defined as a BMI of 19.5 – 24.9 kg/m^2^ whilst a high BMI was defined as a BMI of 25 kg/m^2^ or more. The term “obese” was used to describe all patients with a high BMI whether obese or overweight for simplification. The control samples were obtained from five fertile volunteers with no history of recurrent miscarriage or infertility. Women in the control group were not receiving any form of hormonal therapy and none of them were using an intrauterine contraceptive device. All participants provided written consent to participate in the study and the North of Sheffield Ethics Committee approved the study. Four groups of patients were therefore compared in this study:

1- Obese recurrent miscarriage.

2- Lean recurrent miscarriage.

3- Obese control.

4- Lean Control.

### Investigations for the recurrent miscarriage population

All the subjects underwent investigations according to an established clinical protocol [6], including: prothrombotic studies and antiphospholipid antibody screening, peripheral blood karyotyping, thyroid function tests, thyroid antibodies, androgen profile (total serum testosterone, sex hormone binding globulin and free androgen index), day 2 FSH, LH and oestradiol, transvaginal ultrasonography and hysterosalpingography.

### Endometrial samples

Daily urine testing for Luteinising Hormone (LH) was performed from the mid-follicular phase onwards until the LH surge was detected. Seven to nine days following the LH surge, an endometrial biopsy was obtained with the use of a Pipelle sampler (Prodimed, France). The biopsy was immediately frozen using liquid nitrogen and stored for later processing. All participants used a barrier method for contraception in the biopsy cycle.

### Proteomic analysis

#### Difference gel electrophoresis (DiGE)

Proteins were extracted from endometrial tissue first by grinding in liquid nitrogen with a mortar and pestle, then adding buffer (8 M urea, 5 mM magnesium acetate and 2% CHAPS, 10 mM Tris–HCl, pH 8.5) and grinding further on ice. Protease inhibitor cocktail (Roche complete Mini) was added to the extracts before centrifuging at 15,000 rpm for 15–20 min. Supernatant protein content was assayed using the Coomassie Plus Assay Kit (Pierce Biotechnology). Aliquots of 50 μg of each extract were combined to use as the internal standard in the DiGE experiment. Proteins were labelled using the fluorescent dyes, Cy3 and Cy5, developed for DIGE (GE Healthcare) following the manufacturer’s recommended protocols. Fifty micrograms of protein were labelled with 400 pmol of amine reactive dyes, freshly dissolved in anhydrous dimethyl formamide. Each of the protein extracts was labelled with Cy5 and the pooled internal standard was labelled with Cy3. The labelling reaction was incubated at room temperature in the dark for 30 min and the reaction was terminated by the addition of 10 nmol lysine.

Each Cy5-labeled protein sample (50 μg) was combined with 50 μg of the Cy3-labeled internal standard, and then made up to 450 μl with rehydration buffer (6 M urea, 2 M thiourea, 4% CHAPS). Isoelectric focusing (IEF) was performed using 24-cm Immobiline™ pH 3–10 strips (GE Healthcare) and an Ettan^TM^ IPGphor 3 (GE Healthcare) IEF system. Disulfide bonds were reduced by incubating strips in 1.5 M Tris, pH 8.8, containing 6 M urea, 30% glycerol, 2% SDS, and 1% dithioerythritol. Carbamidomethylation was performed by incubating strips in 1.5 M Tris, pH 8.8, containing 6 M urea, 30% glycerol, 2% SDS, and 4% iodoacetamide. SDS-PAGE was performed using an Ettan^TM^ DALT II (GE Healthcare) and 9-16% gradient gels that were cast using an a2DEoptimizer (NextGen Sciences Ltd). Fluorescent images were recorded using a Molecular Imager FX with PDQuest™ software (Bio-Rad).

#### Image and statistical analysis

Four groups of patients were defined based on the characteristics of weight (lean or obese) and reproductive outcome (control or recurrent miscarriage) (Table 1). Two additional groups were considered by combining all control and all recurrent miscarriage patients (5 and 16 patients, respectively).

**Table 1:** The groups and numbers of patients used in the study

	**Lean**	**Obese**	**Total**
***Control***	3	2	5
***Recurrent miscarriage***	4	12	16
***Total***	7	14	21

Of the possible two-way comparisons of these groups, four were of particular interest in this study – 1) lean control versus lean miscarriage, 2) obese control versus obese miscarriage, 3) all control versus all miscarriage, and 4) lean miscarriage versus obese miscarriage.

Image and statistical analyses were performed using Progenesis SameSpots software (version 3.0 2966.28996) (Nonlinear Dynamics, Newcastle, UK). All images were first warped to a manually selected reference gel. Following warping, the images were grouped into the four experimental groups and changes in spot volumes between different groupings were calculated. All spots showing significant normalized volume changes were manually validated in order to select genuine spots for further statistical analysis. Principal Component Analysis (PCA) was performed using only those spots whose fold changes were statistically significant (p < 0.05).

#### Protein identification

To obtain enough protein in individual gel spots for identification by mass spectrometry, preparative 2D gels containing 1–1.5 mg protein were produced from pooled samples. Protein spots were manually excised from the Coomassie-stained 2D gels and digested overnight at 37°C with sequencing grade, modified porcine trypsin (Promega) in 25 mM ammonium bicarbonate. Peptide tandem mass spectra were obtained using a Bruker ultraflex III MALDI-TOF/TOF mass spectrometer with 4-hydroxy-α-cyano-cinnamic acid (Sigma) as the matrix. Proteins were identified by searching of the mass spectra against the entire National Center for Biotechnology Information Reference Sequence (release 36 with 9,655,479 sequences) using Mascot software (Matrix Science Ltd., London, UK). Search parameters included a maximum of one missed cleavage, a peptide mass tolerance of 250 ppm, and a fragment mass tolerance of 0.5 Da. Only peptide identifications with expectation values above 0.01 are reported.

Finally a Medline literature search was performed without restrictions on date and using a combination of the following key words: proteomics, proteom*, endometrium, endomet*, miscarriage and obesity.

## Results

### Demographics

The main demographic characteristics (age and BMI) of the analysed patient groups and subgroups are summarised in Table 2. The difference between mean BMI values of obese and lean patients is significant (p = 0.01), as expected, whereas none of the other differences were significant. The number of previous miscarriages ranged from 3–6 in the obese group and 3–5 in the non-obese group (p > 0.05). The median number of miscarriages was 3 in both groups.

**Table 2:** The main demographic characteristics (age and BMI) of the analysed patient groups

	**All recurrent miscarriage n** = **16**	**All controls n** = **5**	**p**
*Age*	35 ± 1.3	30 ± 2.1	0.07
*BMI*	26.3 ± 1.6	26.5 ± 2.8	1.0
	**Obese recurrent miscarriage n** = **12**	**Obese controls n** = **2**	
*Age*	33.0 ± 1.6	33.5 ± 2.5	1.0
*BMI*	28.3 ± 1.5	33.0 ± 7.0	0.3
	**Lean recurrent miscarriage n** = **4**	**Lean controls n** = **3**	
*Age*	37.2 ± 1.7	30.0 ± 3.0	0.06
*BMI*	20.0 ± 1.6	23.0 ± 1.0	0.13
	**Obese recurrent miscarriage n** = **12**	**Lean recurrent miscarriage n** = **4**	
*Age*	33.0 ± 1.6	37.2 ± 1.7	0.2
*BMI*	28.3 ± 1.5	20.0 ± 1.6	**0.01**

All values are mean ± SEM.

Twelve of the 16 endometrial samples from the recurrent miscarriage group were obtained from women with unexplained recurrent miscarriage. The remaining four samples (three of 12 samples from the obese miscarriage group and one of four samples from the lean miscarriage group) were from women with a diagnosis of antiphospholipid syndrome.

### 2D gel electrophoresis

**Figure 1:**
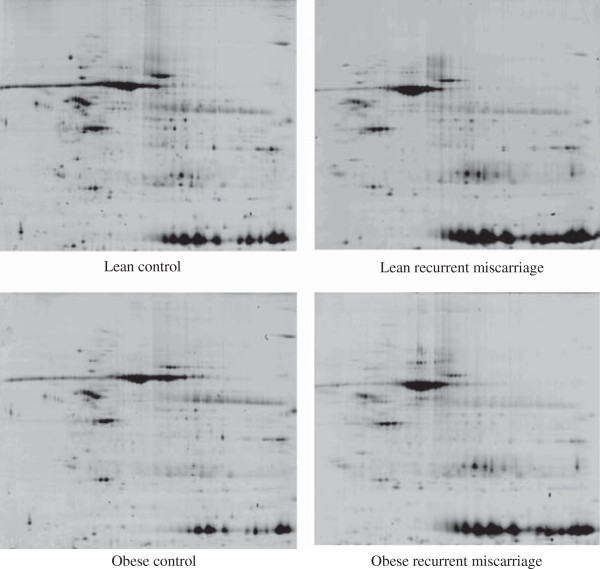
Representative 2-D gel analyses of the four patient groups.

The patient groups were compared by analysis of 2D gels of proteins extracted from endometrial samples. Representative 2D gels from all four patient groups are shown in Figure 1. A total of 544 spots were identified. Protein spots that differed significantly in expression between the groups were used in the PCA to see whether patient groups could be discriminated based on differences in protein expression observed in the 2D gels. The gel spots used for each comparison are shown in Table 3.

**Table 3:** Gel spots used in PCA and protein identities

**Spot number**	**Fold change**	**Direction of change**	**ANOVA (p)**	**Protein identification**	**Mascot score**
	**Recurrent miscarriage *versus *** **control – All subjects**				
1163	2.3	<	0.547	Haptoglobin alpha chain	109
1172	2.2	<	0.547	Haptoglobin alpha chain	*
868	1.8	>	0.547		
254	1.8	>	0.547	Transferrin	547
252	1.8	>	0.547	Transferrin	693
1183	1.6	<	0.547	Haptoglobin alpha chain	*
119	1.6	>	0.547	Serum albumin	162
635	1.6	<	0.547	Haptoglobin beta chain	160
3	1.5	<	0.547	α-1-Antitrypsin	684
489	1.5	>	0.547		
	**Recurrent miscarriage *versus *** **control – Obese only**				
263	5.3	>	0.004		
1172	2.5	<	0.017	Haptoglobin alpha chain	
620	1.9	<	0.025		
1163	2.0	<	0.036	Haptoglobin alpha chain	160
152	1.8	>	0.037		
	**Recurrent miscarriage *versus *** **control – Lean only**				
1163	4.1	<	0.026	Haptoglobin alpha chain	109
901	2.7	>	0.014		
1183	2.2	<	0.036	Haptoglobin alpha chain	*
83	2.0	<	0.030		
252	2.0	>	0.007	Transferrin	693
601	1.9	<	0.048	Hemoglobin beta subunit	522
856	1.9	<	0.020	Hemoglobin beta subunit	167
91	1.6	<	0.049		
561	1.6	<	0.002		
401	1.6	>	0.039		
800	1.6	<	0.020		
184	1.5	>	0.016		
125	1.5	<	0.039		
937	1.5	>	0.023		
659	1.5	<	0.038		
887	1.5	>	0.006		
760	1.4	>	0.019		
1009	1.3	>	0.039		
	**Obese *versus *** **lean – Recurrent miscarriage only**				
1163	2.0	>	0.014	Haptoglobin alpha chain	109
1044	1.8	<	0.010		
930	1.6	>	0.037		
1301	1.5	>	0.040	Transthyretin (Prealbumin)	158
601	1.5	>	0.045	Hemoglobin beta subunit	522

*Identity inferred from position in 2D gel indicating the same molecular weight and nearly the same isoelectric point as spot 1163.

### Principle Component Analysis (PCA)

The results of PCA are summarised in combined plots of the score and loading (biplots) [7]. In these biplots, the coloured dots comprise the score plot, which shows how gels are related to each other based on the intensities of selected gel spots. The colour coding of dots representing gels corresponds to known groupings into which the gels were divided for the analysis. Gels with similar protein expression profiles will be clustered together in the score plot. The numbers in the biplot comprise the loading plot. Each number represents a gel spot and its position in the plot is a measure of its contribution to the clustering of gels.

### Control *versus* miscarriage

**Figure 2:**
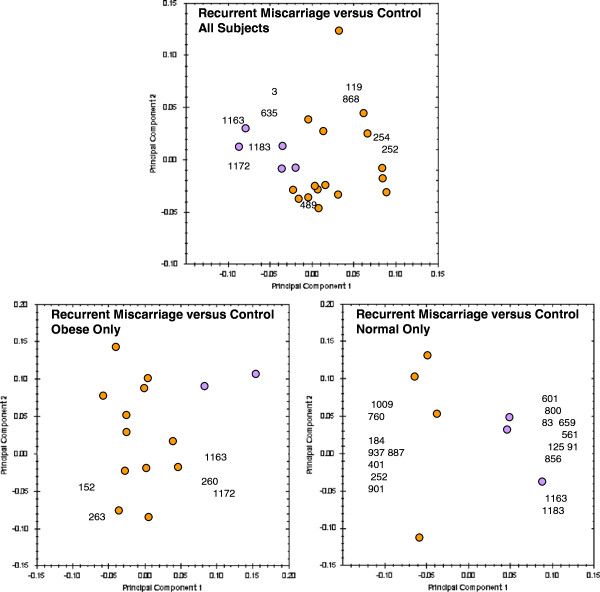
**PCA biplots of recurrent miscarriage *versus *control patient groups.** 2D gels from the recurrent miscarriage groups are represented by orange dots and from control groups by purple dots in the score plot. Numbers in the loading plot indicate gel spots with statistically significant differences in intensity between groups, which were used for the PCA.

Biplots summarising comparisons of patients with recurrent miscarriage and controls are shown in Figure 2. The top panel of Figure 2 shows the comparison of all controls *versus* all miscarriages. These results suggest that protein expression differences between the recurrent miscarriage and control groups are not able to discriminate the two patient groups. The discrimination between recurrent miscarriage and control groups is much better when the obese and lean subgroups are analysed separately (bottom panels in Figure 2).

### Lean *versus* obese

**Figure 3:**
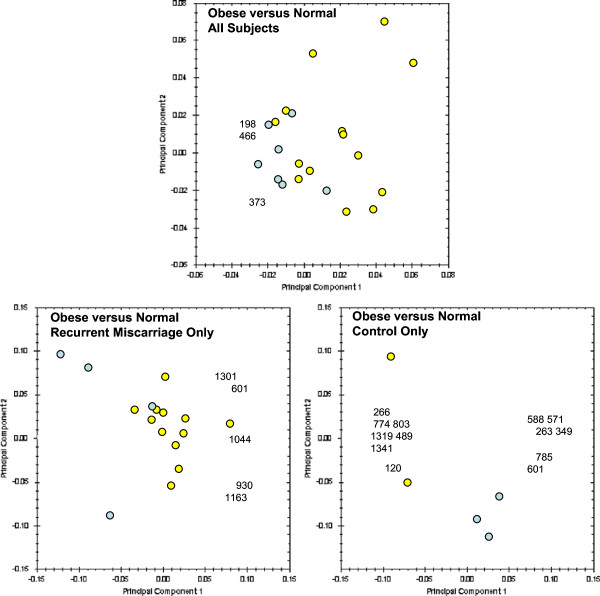
**PCA biplots of obese versus lean groups.** 2D gels from the obese groups are represented by yellow dots and from lean groups by blue dots in the score plot. Numbers in the loading plot indicate gel spots with statistically significant differences in intensity between groups, which were used for the PCA. Plots of all obese versus all lean (top) and obese control versus lean control (bottom right) are included for completeness.

Biplots summarising comparisons of lean and obese patients are shown in Figure 3. Obese and lean patient groups are not well discriminated by the PCA (top panel in Figure 3), but when samples are subdivided based on the presence or absence of miscarriage subgroups show a better discrimination (bottom left and right panels in Fgure 3).

### Mass spectrometry and protein identifications

Gel spots that were included in PCA were excised and the protein in the spot was identified by mass spectrometry of tryptic peptides. Mass spectra were matched to protein sequences using Mascot software, which gives a probability-based score that indicates the likelihood that the match is true. The proteins identified and the corresponding Mascot scores are shown in Table 3. Also shown are the fold differences in intensity for each spot, the statistical significance (ANOVA) of each measured difference, and the direction of the difference (left-most column). All of the proteins identified are serum components. Detection of the same protein in different regions of the gel suggests the presence of isoforms, post-translational modification, and/or degradation.

Haptoglobin alpha chain (spot 1163) appears to contribute to the discrimination observed in all four of the comparisons of interest in this study; the levels of this protein are lower in the miscarriage groups compared to control groups, but higher in the obese miscarriage group compared to the lean miscarriage group.

### Sample size

To better estimate the false discovery rate and address the multiple-testing problem [8], q-values [9] were calculated for each spot. The calculated q-values (Table 3) suggest that about half of the spots determined to have significant fold changes with p < 0.05 could be false positives. The suggested number of samples that would be required in a future study to achieve a desired power of 0.8 [8] for the four group comparisons of interest would be 64 for lean *versus* obese recurrent miscarriage, 30 for lean *versus* recurrent miscarriage obese, 120 for all control *versus* recurrent miscarriage, and 10 for lean control *versus* recurrent miscarriage.

## Discussion

Differential expression of the endometrial protein profile in the different phases of the menstrual cycle has been described by Rai et al. [10] and since then the use of endometrial proteomics has continued albeit mainly concerning the study of endometriosis [11-13] and endometrial cancer [14,15]. To the best of our knowledge, this pilot study is the first to use proteomic analysis to study the endometrial protein expression around the implantation period in women with recurrent miscarriage and to use this technique to investigate the possible effect of increased BMI on the endometrium.

When examining the whole population (obese and lean), the first finding of this study was that PCA had a poor ability to discriminate the samples. Although this may be due to the absence of a distinctive difference in endometrial protein expression between miscarriage and control samples, it may also be due the presence of confounding factors that led to heterogeneity of the samples.We hypothesised that the presence of an increased BMI may be one such confounding factor and repeated the analysis after exclusion of samples from patients with an increased BMI. The analysis was repeated (Figure 2) and PCA then showed good discrimination of recurrent miscarriage and control samples indicating that an increased BMI may indeed have acted as a confounding factor in the initial analysis. Similarly, obese and lean control samples could also be discriminated using PCA, further providing evidence for a difference in endometrial protein expression (Figure 3).

Haptoglobin alpha chain (spot 1163) appears to contribute to the discrimination observed in all the comparisons of interest. The endometrial concentrations of this protein are lower in the miscarriage groups compared to control groups, but higher in the obese miscarriage group compared to the lean miscarriage group. Haptoglobin therefore seems to be affected both by the presence of recurrent miscarriage as well as by increased BMI, as both conditions seem to have an opposite effect on haptoglobin concentrations. Other proteins that were significantly increased in the obese miscarriage group were transthyretin (pre-albumin), and beta globin.

Haptoglobin is a glycoprotein synthesised in the liver. Its primary function is to bind excess haemoglobin thus protecting the kidneys in cases of intravascular haemolysis [16]. However haptoglobin has several other functions that may be relevant to both implantation and obesity.

Firstly haptoglobin is an important component of the body’s response to inflammatory conditions [17-19]. One such inflammatory condition is obesity, where an increase in the central fat compartment leads to a state of relative hypoxia in the adipocytes and a release of a number of inflammatory markers including haptoglobin. Indeed haptoglobin concentrations have been shown to positively increase in proportion to the severity of obesity [17,18]. It is therefore possible that haptoglobin is a marker of an ongoing inflammatory reaction in the endometrial lining of obese women with recurrent miscarriage, which may explain their adverse reproductive outcomes.

Secondly haptoglobin is produced by the endometrium [20] and has been shown in several animal studies to be expressed in increasing amounts in the peri implantation period and is an important component of the extra embryonic matrix [16,19,21]. This increase in endometrial haptoglobin around the time of implantation may play a role in modulating the maternal reaction to the implanting blastocyst [16,19].

The haptoglobin molecule is also known to display genetic polymorphism where some genotypes have been shown to be associated with better reproductive outcomes compared to others [22]. So although obese miscarriage women showed an increased expression of endometrial haptoglobin, it is possible that haptoglobin in this case is of a different genotype that results in a less favourable pregnancy outcome. Future studies should analyse the specific genotype of the haptoglobin molecule using techniques such as Polymerase chain reaction and gel electrophoresis [23].

Obese recurrent miscarriage samples were also associated with a significantly increased expression of transthyretin and beta globin, both of which are common intravascular products normally found in relative abundance in tissue. However, their increased expression in the obese miscarriage cohort may indicate some form of vascular or endothelial dysfunction in these women. Indeed vascular dysfunction is a characteristic of chronic inflammatory conditions such as obesity [24,25].

There are several mechanisms that could support the hypothesis of a local vascular dysfunction in obese women. Firstly hyperleptinaemia has been linked to the occurrence of vascular dysfunction possibly through dysregulation of endothelial nitric oxide [24,25]. Similarly the production of cytokines has also been linked to vascular dysfunction [26]. Finally haptoglobin has been found to have an important role in angiogenesis and vascular dysfunction in chronic inflammatory conditions and this may be more common with certain haptoglobin genotypes [27,28].

It would be interesting to know whether obesity produces any structural changes in fertile women as well as woman with miscarriage and therefore a comparison between obese and non-obese controls would have been ideal. However due to relatively small numbers of the control subgroups, this was not practically possible, but should be addressed in future studies.

A possible confounding factor in this study is the presence of four samples from women with antiphospholipid syndrome which may act as a potential source of bias. Therefore careful consideration was given as to whether these samples should be excluded from the analysis. However the potential effect of antiphospholipid antibodies on pro inflammatory mediators such as tumour necrosis factor α (TNFα) [29] has only been demonstrated in animal studies and recent human studies have been unable to demonstrate similar findings [30]. So even though we cannot rule out a possible confounding effect, it is highly unlikely particularly in view of the small number of women with this condition relative to the whole population.

It was not possible to employ a second analysis technique to confirm the results since all the tissue was consumed in the process of protein extraction and analysis and we were unable to conserve tissue for further analysis. Endometrial samples in this study were obtained in an out-patient setting which results in a relatively small amount of tissue being retrieved and repetition of sampling more than once to obtain more tissue would not have been acceptable due to the associated patient discomfort.

A general limitation of proteomic analysis is that only abundant proteins can be detected. It is possible that other proteins that are relevant to the process of implantation were missed in this analysis either because they were expressed in small amounts or had a molecular weight below the level of detection using our particular method of analysis.

This raises the question as to the best method of approaching the same research question in future studies. Validation of changes in protein expression levels observed with a 2D gel-based approach could be carried out using orthogonal techniques such as Western blotting [31] or a targeted proteomic approach using stable isotope-labeled peptides and multiple reaction monitoring [32]. While the former approach is feasible when appropriate antibodies are available, the latter would require a considerable amount of effort and expense. The problem of abundant proteins in proteomic studies, especially of serum, can be addressed by antibody-based depletion, for example with the Agilent MARS columns. However, a major drawback of using a depletion method is the potential loss of important markers through their non-specific binding to depleted proteins, especially albumin [33]. Other possible approaches include micro arrays and genomic or metabolomic analysis. It is yet unclear whether these technologies would offer an advantage over proteomics when trying to answer the current question.

We would like to emphasise that the results of this study are mainly limited to women with mild obesity; further studies would be needed to examine the effect of more severe forms of obesity on the protein profile. However this may prove difficult since patients with severe degrees of obesity may present more with problems related to infertility rather than recurrent miscarriage. Nevertheless, it may be possible to achieve the necessary numbers in these subgroups in the context of a large multicentre study.

Finally, as there were no previous similar studies, we had no guidance by which to perform a sample size calculation when designing this study. Our findings however have now provided data regarding the necessary sample size for future studies as stated above. Larger studies are now needed to confirm our findings.

## Conclusions

In conclusion the results of this study suggest that Obese and overweight women with recurrent miscarriage have an altered endometrial protein profile that is mainly related to changes in haptoglobin expression. This may provide evidence for an ongoing endometrial inflammatory reaction in the endometrial linings of obese women and may contribute to their higher risk of miscarriage.

## Competing interests

The authors declare that they have no competing interests.

## Authors’ contributions

MM, main investigator, responsible for recruitment of participants, obtaining the samples and writing the manuscript. RP and JT processed the samples, performed the proteomic analysis and assisted with writing the results section. WL and TCL contributed to the study design, interpretation of results, and preparing of the manuscript. All authors read and approved the final manuscript.
